# A Case of Attention Deficit Hyperactivity Disorder in Rhombencephalosynapsis

**DOI:** 10.1007/s12311-021-01234-x

**Published:** 2021-02-15

**Authors:** Dennis J. L. G. Schutter, Marije Paalman, Dylan Henssen, Peter K. H. Deschamps

**Affiliations:** 1 Experimental Psychology, Helmholtz Institute, Utrecht University, Utrecht, The Netherlands; 2 Department of Psychiatry, Utrecht University Medical Centre, Heidelberglaan 100, 3584 CX Utrecht, The Netherlands; 3 Department of Radiology and Nuclear Medicine, Radboud University Medical Centre, Nijmegen, The Netherlands

Sir,

Rhombencephalosynapsis (RES) is a rare disease with a prevalence of 1/1,000,000 likely to be unknown to most child and adolescent psychiatrists and pediatricians. It is a hindbrain malformation characterized with partial or complete agenesis of the vermis, fusion of the cerebellar hemispheres, merging of the dentate nuclei, and continuity of the superior cerebellar peduncles [1]. RES can occur as an isolated phenomenon but also exists in combination with other central-nervous system abnormalities and extra-cerebellar malformations [1]. The aetiology of RES is still unknown. It has been proposed that RES may result from dorsal-ventral organizational defects, which interferes with midline cleavage and subsequently fusion of lateral cerebellar structures during foetal development [3]. Other hypotheses that have put forward suggest the loss of anterior embryonic cells that ultimately form the vermis or from migration defects of these cells to the posterior and/or ventral portions of the cerebellar hemispheres [1]. The clinical presentation and prognosis of RES are variable, but the majority of cases demonstrates impairments in cognitive and affective functions.

Here, we present a case of an 11-year-old boy who was evaluated on several occasions throughout his developmental trajectory to address both psychiatric and somatic problems and we discuss his cerebellar malformation in search of a better understanding of symptom persistence despite an intensive treatment history. He was born at 35 weeks and 4 days, after a dichorionic diamniotic twin pregnancy. After birth, a progressive ventricular dilatation was noted and he received several lumbar punctures after which at 2 months, an adequate liquor balance was reached. Due to red blood cells found in the liquor, a post-haemorrhagic ventricular dilatation was thought to be the most likely cause. Around the age of 6 months, he displayed periods of uncontrollable crying while grasping and rolling his head. In the following months and years, he presented with several somatic problems including strabismus, tremor, stereotypical movements, and gait ataxia. Motor therapy was started to improve motor coordination in late toddlerhood with moderate effects. He maintained a decreased appetite and growth below −2SD in contrast with his twin sister, who showed a typical development without raising concern or need for consultation.

**Fig. 1:**
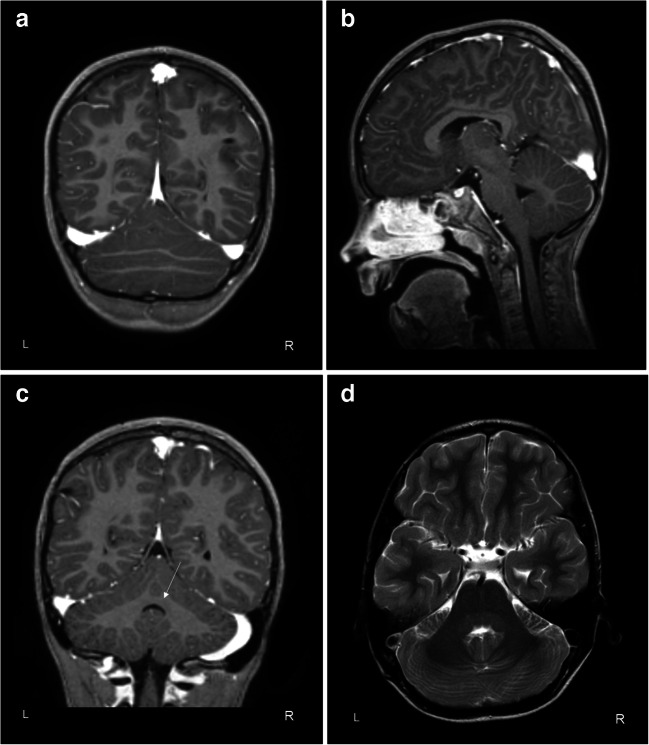
Eight-year-old male patient with rhombencephalosynapsis. Coronal (**a**) and sagittal (**b**) images of the T1-weighted MRI scan show the fused cerebellar hemispheres and absent vermal midline structure. The nodulus (lobule X) is shown on sagittal slice of the T1-weighted MRI scan (**c**) and an axial slice of the T2-weighted MRI scan depicting the deep cerebellar white matter (**d**)

Behaviourally, hyperactivity and issues with emotion regulation were reported as early as from the age of 9 months old. Over infancy and toddlerhood, parents reported an emergence of oppositional behaviour problems. He often had temper tantrums with kicking and beating others and self-harm. As his father had a history of attention deficit hyperactivity disorder (ADHD), his parents recognized the symptoms and he was already referred at the age of 2 for a day-treatment program that included parental support and advice and parent-child interaction therapy. This was followed by special education at the age of 5. At the age of 6, psychiatric and neuropsychological assessment confirmed the diagnosis of attention deficit hyperactivity disorder (ADHD) with symptoms of oppositional defiant disorder and a total IQ of 70. Treatment over the years consisted of several pharmacological options including stimulants (no positive effects, increase in tics and anxiety), risperidone (only temporary effect on aggression), and atomoxetine (moderate improvement of behavioural control). In addition, he received special education, parental advice, and a 3-month period of intensive day-treatment based on a behavioural and systems approach at our clinic. Nevertheless, symptoms of ADHD and ODD persisted. At the age of 8, the boy was hospitalized because of fever, imbalanced gait, headache, and vomiting, and an MRI scan was made. Scanning protocol comprised a T1-weighted post-contrast 1.5-T 3D Sense sequence and a T2-weighted 1.5-T turbo spin-echo sequence. Radiological examination showed the presence of bilateral drain trajectories through the frontal white matter. The gyrus-sulcus pattern of the brain was typical, depicting normal grey-white matter distinction. No abnormalities were observed for myelination rate, neural migration, cerebral midline structures, corpus callosum, basal ganglia, and thalamus. No areas of pathological contrast staining. Normal volume of the lateral and third ventricles with minor asymmetrical appearance. Subtle widening of the temporal horns, but no signs of hydrocephalus. Superficial and deep venous system showed normal filling without signs of thrombosis. In the infratentorial brain, a vermis anomaly with a rostro-caudal gradient and nearly complete fusion of the cerebellar hemispheres was present (Fig. 1a, b). Lingula, lobus centralis, culmen, and declive appeared underdeveloped. Nodulus showed normal configuration (Fig. 1c). The dentate nucleus was indistinguishable from the deep cerebellar white matter (Fig. 1d). The bilateral trajectory of the superior cerebellar peduncle (SCP) appeared typical and no evidence of fusion caudal to the decussation of the SCP or nucleus ruber. Morphology of the folia was normal and cerebellar volume (137 cm^3^) comparable to healthy controls aged between 10 and 13 years (*n*=35, mean ± SD, 138 ± 14 cm^3^, *t*= 0.57, *p*= 0.57, two-tailed). Exome sequencing and a single nucleotide polymorphism array revealed no DNA abnormalities that provided a genetic basis for our observations.

The psychiatric profile of our patient concurs with prior case reports of patients with RES that observed psychiatric symptoms including stereotypical behaviours, aggression, self-injurious behaviour, restlessness, and impulsivity [2]. Moreover, the boy’s behaviour fits the constellation of behavioural control disturbances in patients with isolated cerebellar lesions, including hyperactivity, impulsivity, irritability, emotional disinhibition, and aggression [4]. The symptoms reported in the scientific literature supports the idea that the cerebellum is important for behavioural control and part of a dysfunctional brain network in ADHD as indicated by volumetric reductions of the cerebellar vermis and behavioural parent ratings of ADHD in medication-naïve adults [5, 6]. In addition, previous studies have linked partial RES and other abnormalities of the vermis to symptoms associated with stereotypical behaviours and limited social skills, as well as impaired executive functions and emotion regulation, as seen in children with autism spectrum disorders (ASD) [7–9]. As children with ADHD-related symptoms often show ASD symptomatology and there is substantial overlap of symptoms in both disorders [10], the cerebellum may present a shared neural correlate. Of note, internalizing behaviours like obsessive thoughts and compulsive actions in conjunction with abnormalities of the vermis have also been observed, hinting towards an even broader role for cerebellar neural correlate in psychiatric syndromes [2].

Our own observations are consistent with a general cerebellar cognitive affective syndrome that is characterized by non-motor deficits in the cognitive and affective domain related to acquired and congenital lesion of the cerebellum [11]. Specifically, damage confined to the vermis is suggested to be associated with impairments in the experience and regulation of emotions.

RES often goes accompanied by supratentorial forebrain pathology that can include hydrocephalus, holoproencephaly, absent septum pellucidum, corpus callosum dysplasia, and malformations of the anterior commissure, temporal cortex, and mammillary bodies [1]. Interestingly however, our patient does not show obvious signs of supratentorial pathology. So, the fact that the abnormalities in our patient are mainly confined to the cerebellum, we propose that the behavioural disturbances in our patient result from damage to the vermis and its deep cerebellar fastigial nuclei that project to the brainstem reticular nuclei and forebrain regions [4].

In conclusion, our findings suggest that cerebellar pathology is involved in the treatment-resistant psychiatric symptoms associated with emotion dysregulation. The presence of cerebellar abnormalities may be considered by child psychiatrists and neurologists especially when infantile psychiatric disturbances are associated with neurological, cognitive, affective, and somatic manifestations. Our case report adds to the growing evidence on the importance of the cerebellum in cognitive and affective symptoms in psychopathological conditions, and raises the question of differential treatment effects when cerebellar pathology is present.
